# Comet assay and antioxidant enzyme as blood biomarkers of low-dose radiation-induced adaptive response

**DOI:** 10.1038/s41598-026-60634-3

**Published:** 2026-07-16

**Authors:** Misara M. Awad, Ahmed A. Abdelrahman, Ahmed A. Abdel-Aal, Ibrahim M. Hassan, Seham M. El-Marakby

**Affiliations:** 1 Physics Department, Faculty of science, Al-Azhar University, Cairo, Egypt; 2 Biophysics lab, Radiation Physics Department, National Center for Radiation Research and Technology (NCRRT), Egyptian Atomic Energy Authority (EAEA), Cairo, Egypt; 3 Basic Science Department-Faculty of Energy Sciences, Benha National University, Obour City, Cairo, Egypt

**Keywords:** Biochemistry, Biomarkers, Medical research, Molecular biology

## Abstract

This study investigated the ability of low doses to cause an in vivo radio-adaptive response (RAR) and examined the biological effects of acute low-dose total-body gamma irradiation in rats. Adult male rats were randomly divided into 8 groups. Acute gamma radiation doses of 0.25, 0.5, and 0.75 Gy for groups 2, 3, and 4, respectively, and a 2 Gy challenge dose for group 5 were administered 14 days later. then 0.25, 0.50, and 0.75 Gy, followed by 2 Gy to groups 6, 7, and 8. Twenty-four hours after irradiation, measurements were made of comet assay parameters and antioxidant indicators: Acute gamma exposure resulted in a significant rise in Comet Assay Score CAS (6–16%), Tail Length TL (4.7–7.45 μm), DNA in tail DNA% (11.82–17.76%), Tail Moment TM (1.17–3.78 μm), and Olive Tail Moment OTM (0.53–1.52 µmol/L), as well as dose-dependent increases in GSSG (4.6–9.58 µmol/L), and decrease in SOD (180.4–91.2 U/mL). These results demonstrate the rising detrimental consequences linked to higher acute gamma doses. Lower priming does encourage stronger adaptive responses, according to RAR analysis. The concentrations of GSSG, TL, OTM, and the ratio of Oxidized Glutathione/ Reduced Glutathione GSSG/GSH showed notable linear dose-response associations, indicating their potential as biomarkers for acute gamma radiation biodosimetry. These findings lay the groundwork for future research to verify these markers for dose evaluation and to examine the dynamics of RAR under various challenge doses and postirradiation circumstances.

## Introduction

Ionizing radiation’s long-term consequences are mostly known, although the precise risks and specifics in various groups are still not entirely understood. To understand how cells respond to ionizing radiation exposure, it is crucial to comprehend how cells react to it to create prediction indicators that may be used to evaluate human exposure. Many molecular markers can be used to evaluate radiation exposure to humans. FDXR, CDKN1A, and PCNA genes are among the many molecular biomarkers that have been observed at 1–3-fold higher levels in samples exposed to 0.1–1 Gy of X-rays^1^. Amino acid metabolism, fatty acid metabolism, dyslipidemia, and lipid peroxidation all point to an inflammatory phenotype with potential long-term effects on general health following exposure to modest doses of high linear energy transfer (LET) radiation exposure^2^. Following this radioactive incident, biomarkers to track possibly exposed populations is expected to be highly beneficial. To track occupational or environmental radiation exposure and forecast a patient’s response to radiation therapy, recent research has looked for predictive markers of intrinsic radiosensitivity in healthy people^3^. Cytogenetic methods are the most popular indicators of exposure^4^. Conventional cytogenetic assay-based radiation exposure biomarkers take a long time to produce results. For biomonitoring of human populations, the micronucleus (MN) assay is often used^5^. Some of these recent methods allow accurate assessment of low-dose ionizing radiation effects, including evaluation of DNA damage, oxidative stress, and adaptive responses^6,7^. Since apoptotic and epigenetic processes occur continuously in healthy individuals exposed to background radiation, it is challenging to identify specific biomarkers of cytotoxic (apoptotic) and epigenetic changes induced by low-dose ionizing radiation. Changes in gene expression have been proposed as a potential biomarker for radiation biodosimetry^8^. The difficulty in assessing biodosimetric parameters of nuclear exposure and in performing effective triage of radiation-exposed populations still requires further improvement. Radioactive accidents demand better and practical monitoring tools to enhance radioprotection. In particular, the development of reliable biomarkers for dose estimation would be useful for assessing human health risks associated with low-dose exposure. In the event of a nuclear accident, the importance of monitoring prophylactic and therapeutic doses will differ significantly because radiation shielding is typically necessary. The creation of suitable biomarkers and dose analyses is essential to solving this issue^9^.

Exposure to ionizing radiation can cause DNA damage and can be detected at the single-cell level using single-cell gel electrophoresis, also known as the comet assay^10^. This method involves embedding single cells in agarose, followed by cell lysis, electrophoresis, microscopy, and image analysis. To facilitate the use and analysis of comet assay parameters, computer software has been developed^11,12^. Several modifications have been introduced to the comet assay to facilitate the detection of DNA damage, including inter-strand breaks^13^. The sensitivity and speed of the comet assay have attracted interest in its use as an indicator of radiation exposure. These biomarkers have been studied to assess radiation exposure and its biological effects in uranium miners^14^. Significant increases in DNA damage have been observed when comet assay parameters were used as biomarkers for medical assessments exposed to ionizing radiation at work^15^. Similar research has been conducted on nuclear workers exposed to ionizing radiation for extended periods^16^.

Exposure to ionizing radiation disrupts the delicate balance between endogenous antioxidant defense mechanisms and the overproduction of reactive oxygen species (ROS), thereby inducing oxidative stress. Under physiological conditions, cellular redox homeostasis is maintained by a coordinated antioxidant network comprising enzymatic defenses, including glutathione peroxidase (GPx), Catalase (CAT), and Superoxide Dismutase (SOD), as well as non-enzymatic antioxidants such as reduced glutathione (GSH). Together, these systems efficiently neutralize ROS generated as normal metabolic byproducts, thereby preserving the structural and functional integrity of proteins, lipids, and nucleic acids^17^. According to Lobo et al.^18^ and Valko et al.^19^, ionizing radiation greatly boosts the production of ROS, overriding these defense mechanisms and causing oxidative damage. Reduced activity of endogenous antioxidant enzymes is a key sign of oxidative stress; for instance, reduced activity of SOD, CAT, or GSH has been linked to increased levels of free radicals after radiation exposure. According to Ayala et al.^20^, elevated levels of malondialdehyde (MDA), a byproduct of lipid peroxidation, indicate higher oxidative damage to cellular membranes. Oxidative stress is further exacerbated by oxidized glutathione (GSSG), hydrogen peroxide H_2_O_2_ reduction using GSH as a substrate, and GPx depletion with H_2_O_2_ buildup^17^. Therefore, alterations in particular redox metabolites and antioxidant biomarkers can function as sensitive markers of radiation-induced oxidative imbalance^21^. Oxidative stress results from H_2_O_2_ oxidizing GSH to GSSG^22^. Although fluorescent probes like dihydrorhodamine and mitochondria-targeted dyes like MitoSOX for cellular and mitochondrial ROS, as well as DAF-2 for nitric oxide detection, can be used to measure ROS directly and provide sensitive real-time assessment of oxidative stress in biological systems^18^. In this study, we used indirect measures such as antioxidant enzyme activity.

Radiation adaptive response (RAR), a biological mechanism which is initiated when an organism is exposed to low or moderate doses of genotoxic insults such as ionizing^23^ and non-ionizing radiation^22^. Through this process, defense mechanisms are induced, such as enhanced DNA repair, allowing the organism to withstand subsequent higher-dose exposures^24,25^. Although RAR can appear in a variety of experimental settings, the priming dosage effect also known as the Raper-Yonezawa effect is the most common and straightforward. In this case, RAR is triggered by an initial low dose (referred to as an “adaptive,” “priming (PD),” or “conditioning dose”)^26,27^. It is observed when this initial small dose, after a certain time interval, is followed by a higher challenging dose (CD), resulting in smaller genotoxic effects than those expected from the challenging dose alone^28^. Constant dose-rate irradiation, in which adaptive signals saturate after a specific amount of time, is another potential RAR form^29^. Various physical and chemical agents have been documented to cause RAR in a range of organisms, including bacteria^30^, and human cells^31–33^. Several biological indications (endpoints), such as DNA damage, chromosomal abnormalities, cell viability, survival rates, and mutation rates, were evaluated to accurately characterize this response. AR’s potential for radiation protection and cancer treatment, particularly in radiotherapy, is now under investigation^33^. According to estimates, RAR manifests in 50–78% of priming-challenging dose situations^34^ and only in 45% of constant dose-rate populational investigations of RAR, making this inquiry extremely important^27^. Additionally, recent research has demonstrated that low-dose radiation exposures can improve cellular stress tolerance and modify oxidative stress pathways with detectable alterations in antioxidant system activity, indicating a connection between adaptive responses and reactive oxygen species (ROS) dynamics^35^. AR strongly influences the response of living organisms to genotoxic insults and may provide new ways to counteract such damage by precisely triggering and regulating the adaptive process^33^. The present study aims to investigate the role of antioxidant enzymes in RAR and to explore potential applications of adaptive responses in biological systems.

This study sought to answer the following questions by examining the effects of acute gamma radiation exposure at low and moderate levels of 0.25, 0.50, and 0.75 Gy on a few biophysical features of rat blood: Does acute low-dose gamma radiation exposure pose a risk? Does exposure to a low priming dose of gamma radiation prior to a high dose of the same type of radiation cause RAR? Which parameters could be used as biological dosimeters for acute gamma radiation exposure? In this study, we established two new terms, the Radio-Adaptive-Response Factor (RARF) and the Radio-Adaptive-Response Equivalent Dose (RARED), to characterize the average changes in numerous biological parameters following an acute priming dose administrated in rats subjected to various high doses of irradiation. This factor allows for the measurement of biomarker changes caused by a radio-adaptive response dose. This new metric improves the interpretability of adaptive responses by quantifying and measuring biomarker changes brought on by a radio-adaptive response dose.

## Materials and methods

### Animal preparation

For the tests, adult Wister (6–7 weeks old) male albino rats weighing 120 ± 10 g were acquired from the Rat House at the National Research Center in Egypt. The animals were given regular rat diet that was balanced and contained roughly 21% protein, 5% fiber, 3.5% fat, and 6.5% ash. The animals are kept in an animal home with free access to food and water, a controlled temperature of 26 °C, and a 12-hour day/night cycle of light.

### Animal exposure to acute gamma radiation

The animals were divided into eight groups at random, with six rats in each experimental group. Group 1 was the sham-irradiated control group. The rats in this group were kept in identical cages as those that were exposed to gamma radiation for the same amount of time, but without radiation. Rats in Groups 2 and 6 received a total gamma priming dose of 0.25 Gy of acute gamma radiation. Group 6 received an additional challenge dose of 2 Gy after 14 days. Rats in Groups 3 and 7 were given a priming dose of 0.5 Gy of acute gamma radiation, and Group 7 was given an additional challenge dose of 2 Gy. Rats in Groups 3 and 8 received a total gamma priming dose of 0.75 Gy of acute gamma radiation. Group 8 received an additional challenge dose of 2 Gy after 14 days. Rats in Group 5, which used as the RAR control group, only got a 2 Gy dose. The rats were gathered for analysis 24 h following irradiation. The final analysis included every animal. The animal irradiation plan is summarized in Table 1.

### Declaration of animal treatment

All animal procedures were approved by the Research Ethical Committee for the National Center for Radiation Research and Technology of REC-NCRRT have Approval No: F/51A/25. The study adhered to the 3Rs principles and complied with CIOMS and ICLAS International Guiding Principles and all relevant institutional, national, and international regulations for the care and use of laboratory animals. The work was conducted and reported in accordance with ARRIVE guidelines.

### Description of irradiation setup

The National Center for Radiation Research and Technology (NCRRT), Atomic Energy Authority, Cairo, Egypt, used the Canadian Gamma Cell-40 irradiator (¹³⁷Cs source), manufactured by Atomic Energy of Canada Ltd., Ontario, Canada, to perform whole-body gamma irradiation. A reference alanine dosimeter traceable to the National Institute of Standards and Technology (NIST, USA) yielded an equivalent exposure rate of 0.695 Gy/min for all classified rat groups. In accordance with ASTM E 1026 procedures, the irradiator was previously calibrated using a Fricke reference standard dosimeter. To ensure that every animal received the same dosage, samples were placed in the middle of the irradiation chamber at 35 cm from the source. The chamber’s purpose is to reduce dosage heterogeneity brought on by sample mobility. Animals can be consistently oriented in relation to the gamma source thanks to the turntable and platform arrangement. To ensure that every sample received the desired exposure, dosimetric mapping was used to confirm the distribution of the dose within the chamber. To ensure reproducibility and precision of the administered gamma radiation, all irradiation processes were carried out in accordance with quality assurance standards, including routine verification of the dose rate, chamber geometry, and sample positioning. This configuration enables accurate whole-body exposure while preserving radiation dose uniformity among all rat patients^36^.

**Table 1:** Irradiation plan to different gamma doses for all classified groups.

Group	Description	Dose rates (0.695 Gy/minutes)	Period between two irradiations (days)	Challenge dose (Gy)	Time before measurements (Hours)
G1	Control (sham)	0	14	0	24
G2	Low priming dose	0.25	14	0	24
G3	Moderate priming does	0.5	14	0	24
G4	Higher priming dose	0.75	14	0	24
G5	Challenge does only (RAR control)	0	14	2 Gy	24
G6 (P + C)	Low Priming + Challenge dose	0.25	14	2 Gy	24
G7 (P + C)	Moderate Priming + Challenge dose	0.5	14	2 Gy	24
G8 (P + C)	Higher Priming + Challenge dose	0.75	14	2 Gy	24

### Preparation of blood samples and analysis

Blood samples were collected via retro-orbital sinus puncture under anesthesia with 1.2 g/kg urethane (I.P.) to minimize discomfort^37^. Upon completion of the experiments, animals were humanely sacrificed and the absence of corneal reflexes and breathing confirmed euthanasia^38^. Collected blood was centrifuged at 3000 rpm for 10 min to obtain serum, which was stored at − 20 °C until biochemical analyses, including lipid and liver profiles, were performed.

### Biochemical examination

For the biochemistry analysis, the serum was Aliquoted into different tubes devoid of anti-coagulant ingredients. Before measurements, the sera were centrifuged for 10 min at 3000 rpm and refrigerated at − 20 °C for nearly a week. By evaluating the catalytic reduction of hydrogen peroxide, the Sinha^39^ method was used to calculate catalase activity (CAT). The Minami and Yoshikawa^40^ method was used to measure the superoxide dismutase (SOD) activity. However, Ahmed et al.^41^ used this technique to quantify the amount of (GSH). Wu et al.^42^ described the use of an enzymatic recycling test to assess GSSG levels after masking GSH. Oxidative stress was measured using the GSSG/GSH ratio.

### Comet assay score analysis

With modifications and thorough reporting in compliance with the Minimum Information for Reporting on the Comet Assay (MIRCA) guidelines outlined by Møller et al. (2020)^43^. in Nature Protocols, which offer crucial standards for characterizing comet assay methods and outcomes in scientific investigations, the alkaline comet assay was carried out as previously reported by Singh et al.^44^. Comet Assay Score (CAS), Tail Length (TL), Tail Moment (TM), percentage of DNA in the tail (DNA%), and Olive Tail Moment (OTM) were among the comet assay outcomes assessed. The high metabolic activity and known vulnerability of hepatic tissue to radiation-induced genotoxic stress led to its selection. Following euthanasia, about 20 g of liver tissue was taken, washed with ice-cold phosphate-buffered saline (PBS), and centrifuged for 15 min at 4 °C at 4000 rpm. As advised for reporting, every step from tissue collection to cell embedding was finished on ice in less than 30 min to reduce artifactual DNA damage. Isolated hepatocytes were embedded in low-melting-point agarose to preserve DNA integrity. On sterile absorbent paper, the resultant pellet was lightly dotted. Low-melting-point agarose was used to implant hepatocytes. Low-melting-point agarose was made with cells at a final concentration of around 0.7% (wt/vol), which is considered a crucial criterion for protocol reporting. Agarose (Solarbio, Beijing, China) and cells (~ 1 × 10) were dispersed in a 2-gel configuration on precoated glass slides. To eliminate cellular all soluble cell components and membranes leaving DNA in the form of nuclei, slides were immersed in a pH 10 lysis solution at 4 °C for 60 min after solidification. A Nikon fluorescence microscope (Tokyo, Japan) was used to take fluorescent pictures at a magnification of 20×. CASP software (version 1.2.3 beta 1; Casp-Lab Comet Assay Software Project) was used to score comets. In accordance with the MIRCA guidelines, a minimum of fifty randomly chosen comets per slide were blindly assessed for each treatment group, and the software version and imaging magnification were recorded. All slides were coded before processing and decoded immediately after scoring was finished to prevent observer bias. For efficient cell lysis and minimal DNA damage during processing, the lysis solution comprised 2.5 M NaCl, 100 mM EDTA, 10 mM Tris, 1% Triton X-100, and 10% DMSO. ⟯.” To unwind the DNA, slides were then treated for 20 min in alkaline electrophoresis buffer (300 mM NaOH, 1 mM EDTA, pH > 13). In accordance with the MIRCA checklist, the electrophoresis was carried out for 30 min at a field intensity of approximately 1.0 V/cm (~ 25 V over a 25 cm tank platform) at 4°C. The temperature, time, chemical composition of the buffer, and electrical field strength (V/cm) were recorded as critical parameters. Slides underwent three 5 min rinses in 0.4 M Tris buffer (pH 7.5) to neutralize them after electrophoresis. Ethidium bromide (5 mg/L; Sigma, St. Louis, MO, USA) was used to stain the slides for 10 min in the dark. The DNA dye, ethidium bromide, is a crucial reporting parameter in comet assay investigations.

### RAR equivalent dose (RARED) and RAR factor calculation

The term radio adaptive response equivalent dose (RARED, D_RAR_) introduces a quantitative measure of the equivalent acute radiation dose corresponding to the observed biological changes induced by radioadaptation. In brief, RARED represents the theoretical radiation dose that would produce a comparable biological effect in the absence of prior adaptive exposure, thereby enabling the assessment of the organism’s ability to tolerate a subsequent challenge dose. In the present study, RARED was determined by solving the inverse of the fitted dose–response function, i.e., identifying RARED (D_RAR_) such that:

f (D_RAR_) =Y_adaptive_. It should be emphasized that D_RAR_ represents an equivalent biological dose rather than the physically administered dose. Therefore, although the combined priming and challenge doses reached 2.25–2.75 Gy, the corresponding D_RAR_ values may remain substantially lower and within the experimentally measured acute dose range.

The RAR factor ($\:{f}_{RAR}$) was defined as a normalized dose-based metric quantifying the relative reduction in effective dose due to radioadaptation, calculated from the difference between $\:{D}_{RAR}$ and the priming dose ${D}_{p},$ scaled to the total delivered dose ${D}_{p+c},$, as expressed in Eq. (1).

1 $$\:f_{{RAR}} = 1 - \frac{{(D_{{RAR}} - D_{p} )}}{{D_{{p + c}} }}$$

The inclusion of the priming dose term (Dp) reflects the biological role of the priming exposure in initiating the adaptive response. Equation (1) was developed following the same general principle as the Yonezawa effect scheme^45^, known as “the priming dose model”, which quantifies the magnitude of radioadaptive protection relative to a reference condition, as expressed in Eq. (2)^46^.

2 $$\delta = 1 - \frac{{N_{{p + c}} }}{{N_{c} }}$$

where $\:\left({N}_{c}\right)$ is the marker reading following the challenge dose without the priming dose. and $\:{(N}_{p+c})$ represents the marker reading following combined priming and challenge doses.

The RAR factor was calculated only for groups exhibiting a valid equivalent dose (D_RAR_) within the experimentally measured acute dose range. Consequently, the present formulation is intended to quantify the magnitude of an established radio adaptive response rather than to determine the presence or absence of adaptation. Groups for which no valid $\:{D}_{RAR}$ solution could be obtained within the calibrated dose range were classified as non-adaptive and excluded from RARF estimation. Higher $\:{f}_{RAR}$ values indicate stronger adaptive protection, with values approaching unity corresponding to the highest adaptive effect observed within the valid calculation range. This formulation is conceptually analogous to the classical Yonezawa parameter (δ), which is based on the priming dose model, which evaluates the relative change in biological endpoints according to Eq. (2). However, in the present work, the formulation is expressed in terms of equivalent dose rather than direct biological readouts. All $\:{D}_{RAR}\:$values were obtained by inverse interpolation within the experimentally calibrated and monotonic region of the fitted acute dose–response relationship. A valid RARED value was accepted only when the estimated equivalent dose ($\:{D}_{RAR}$) fell within the experimentally measured acute dose range. Each data point represents the mean ± SD of five independent measurements per dose level.

### Statistical analysis

In addition to standard deviation (SD) values, we computed the average outcomes of five rats in each group during our statistical analysis. Additionally, we calculated the threshold of significance for *p* ≤ 0.05, where the variation satisfies the tolerance conditions and is deemed significant. We used Origin Pro 2021b program developed by OriginLab (https://www.originlab.com/*)*, to fit all the data. For every parameter of acute gamma doses to radiation (0.25, 0.5, 0.75, and 2 Gy), as well as their reverse functions, this includes calibration functions and dose-response correlations.

## Results

### Antioxidant enzymes for the blood

**Fig. 1:**
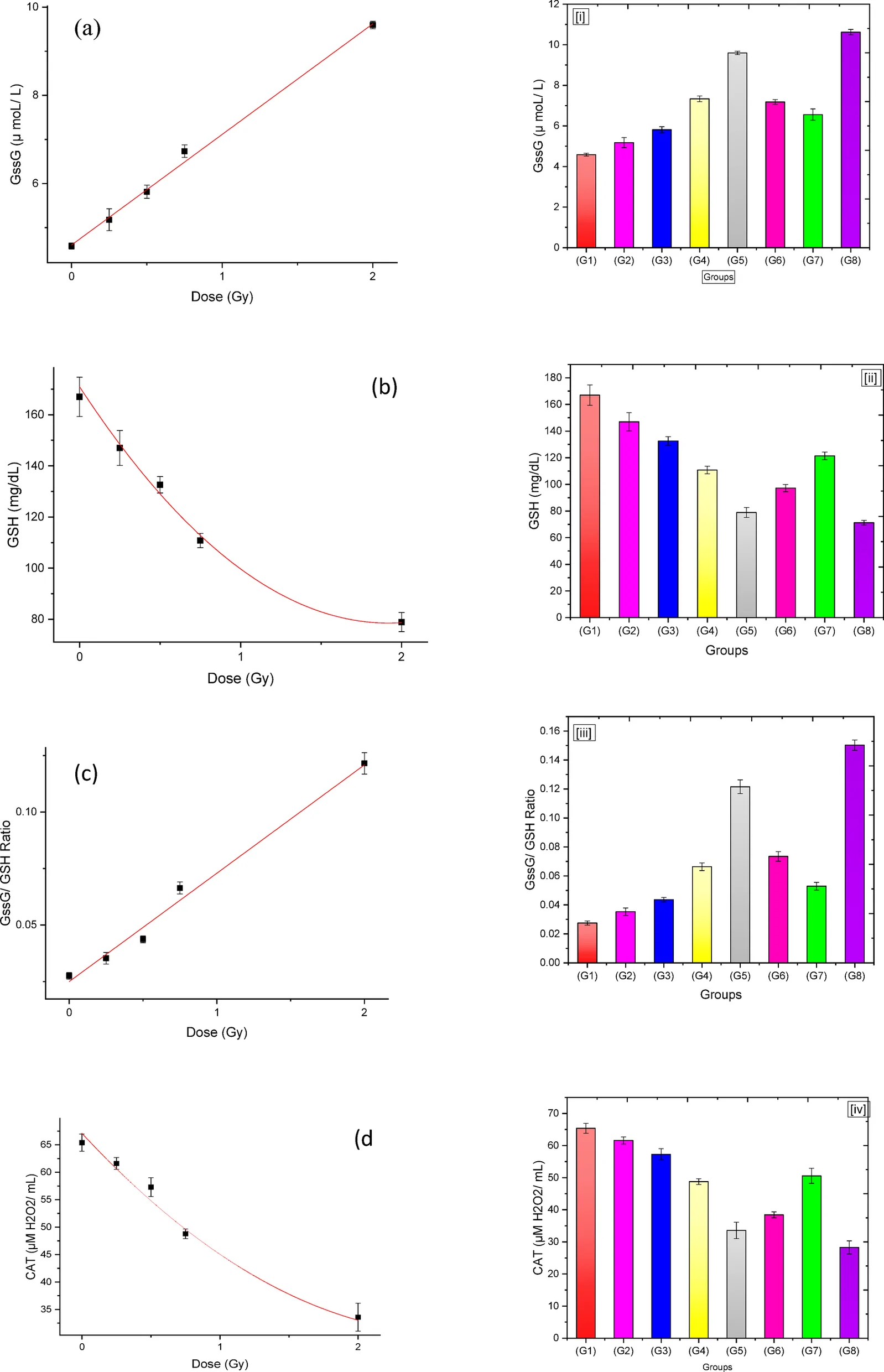

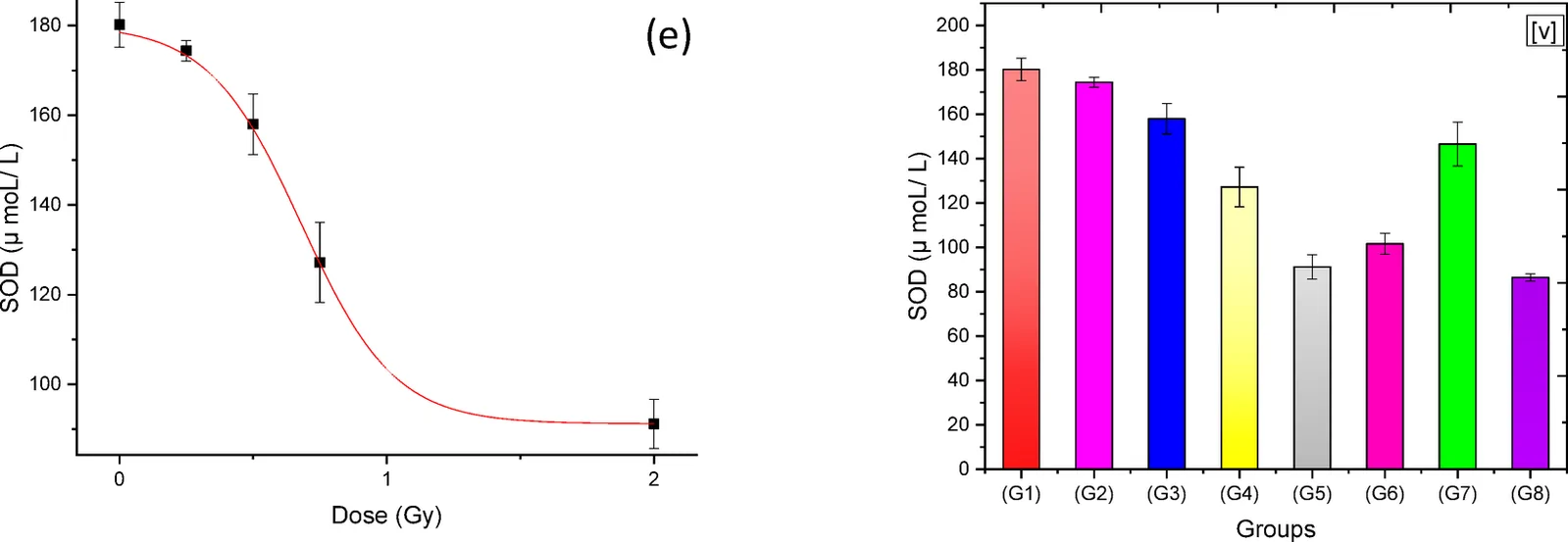
Effects of different acute gamma radiation doses (0, 0.25, 0.50, 0.75, and 2 Gy) on antioxidant enzymes across the five groups (G1–G5). Panels (**a**–**e**) represent [GSSG], [GSH], GSSG/GSH ratio, [CAT], and [SOD], respectively. Panels (**i**–**v**) illustrate the overall trends of these parameters across all groups.

The Figure 1 shows how acute gamma radiation affects important antioxidant indices such as, GSSG, GSH, CAT and SOD. At the same time, compared to controls, GSSG levels and the GSSG/GSH ratio increased significantly linearly across acute radiation doses (Table 2), increasing by 13.00–109% and 26.89–343%, respectively. On the other hand, GSH levels significantly decreased in a polynomial pattern, dropping from 53 to 11.98% in comparison to G1. CAT is fitting parabola and SOD is dose-response fitting drop in rats given acute doses of 0.25, 0.50, 0.75, and 2 Gy (G5), with reductions ranging from 5.81 to 49%, and 3.33 to 47% respectively, in comparison to the control group (G1), suggesting significant inhibition of these enzymes. Antioxidant enzyme levels were significantly greater in the 0.5 + 2 Gy group (G7) for the radioadaptive response (RAR) groups. GSSG decreased by 38% when comparison to G5, While GSH, CAT, and SOD increased by 53.11%, 50.64%, and 60.09%, respectively, while GSSG fell by 33.75% in comparison to G5. GSSG dropped by 25.18%, GSH, CAT, and SOD increased by 23.35%, 14.45%, and 10.75%, respectively, in G6, showing moderate but statistically significant improvements over G5. GSSG was the most informative marker for assessing RAR and showed the highest sensitivity to acute gamma radiation among the enzymes examined. It is interesting to note that the 0.75 + 2 Gy (G8) group showed decreases in all antioxidant measures when compared to G5, including GSH (− 10.44%), CAT (− 15.77%), SOD (− 5.18%), and indicating ongoing oxidative stress even after previous adaptive exposure.

**Table 2:** Fitting parameters for the relationship between antioxidant enzyme levels and gamma radiation dose in groups exposed to an acute gamma radiation dose, including the control group (zero dose).

Enzymes	Type of fitting	Equation	Equation constants	*R*-value	Significance
GssG	Linear	* y=ax+b*	$\:a=\:4.61\pm\:0.060$ $\:b=\:2.51\pm\:0.056$	0.99848	2.30$\:\times\:{10}^{-6}$
GSH	Polynomial	$\:y={intercept+B}_{1}X$ $\:+{B}_{2}{X}^{2}$	Intercept =$\:170.99\pm\:\:6.17$ $\:{B}_{1}=-96.44\pm\:13.94$ $\:{B}_{2}=\:25.142\pm\:5.74$	0.98934	$\:1.06\times\:{10}^{-2}$
GssG/GSH ratio	Line	* y=ax+b*	$\:a=\:0.025\pm\:0.003$ $\:b=\:0.048\pm\:0.113$	0.958	$\:7.66\times\:{10}^{-4}$
CAT	Parabola	$\:y\:=\:A\:+\:B*x\:+\:C*x^2$	$\:A=67.09975\:\pm\:\:1.94266$ $\:B=-27.08444\:\pm\:\:5.19868$ $\:C=5.02468\:\pm\:\:2.67472$	0.97535	* 8.04564E-4*
SOD	DoseResp	$\:y=\:{A}_{2}+\frac{({A}_{2}-{A}_{1})}{\left({10}^{(\left(LOGxo-x\right)\times\:p}\right)}$	$\:A1=91.2\:\pm\:\:3.30022$ $\:A2=180.2\:\pm\:\:4.01239$ $\:\mathrm{L}\mathrm{O}\mathrm{G}\mathrm{x}0=\:0.681\pm\:\:0.038$ $\:P=-2.5\:\pm\:\:0.57524.$	0.9984	* 0.0098*

### Double and single strand DNA breaks on rats using single cell gel electrophoresis (Comet Assay)

**Fig. 2:**
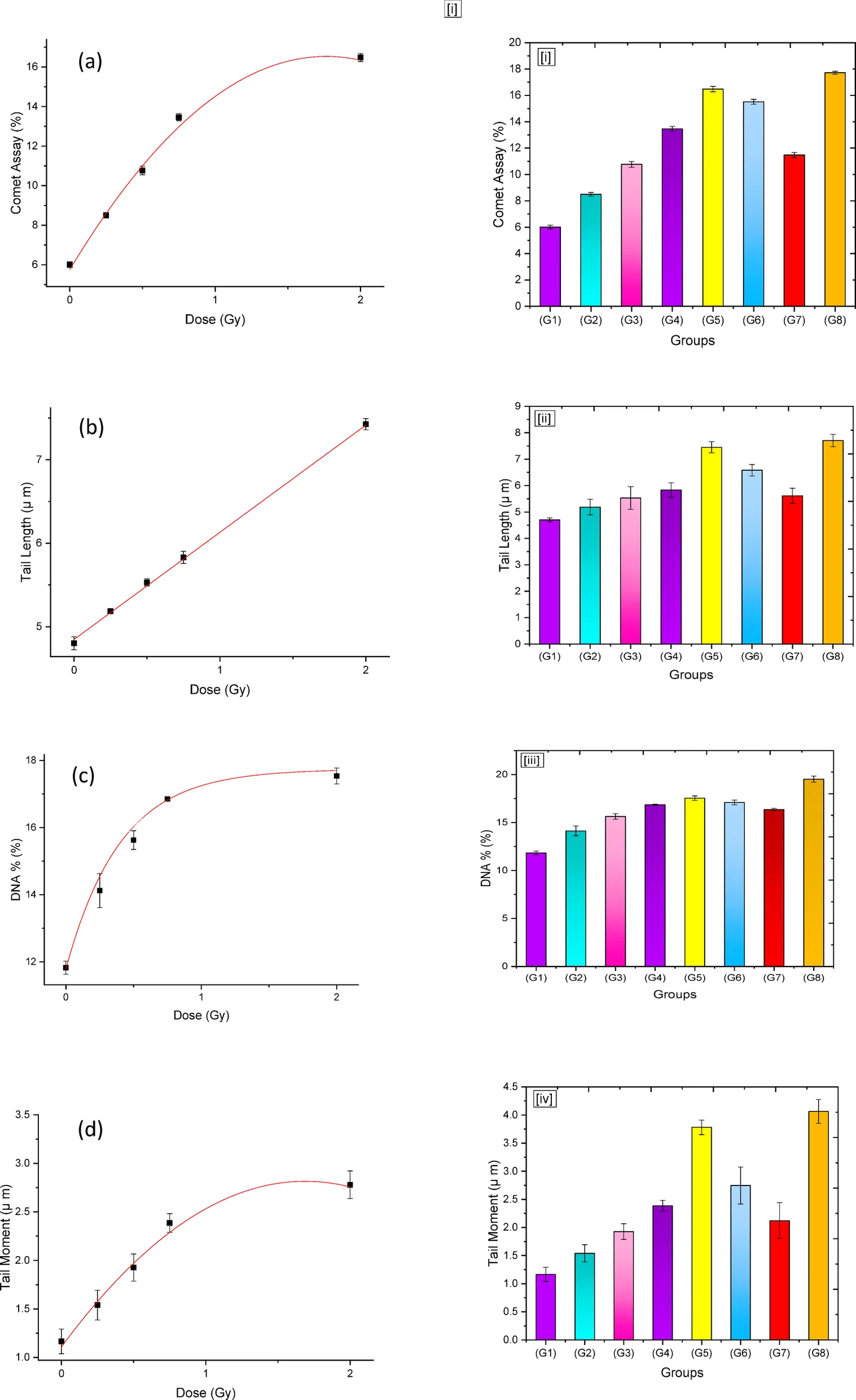

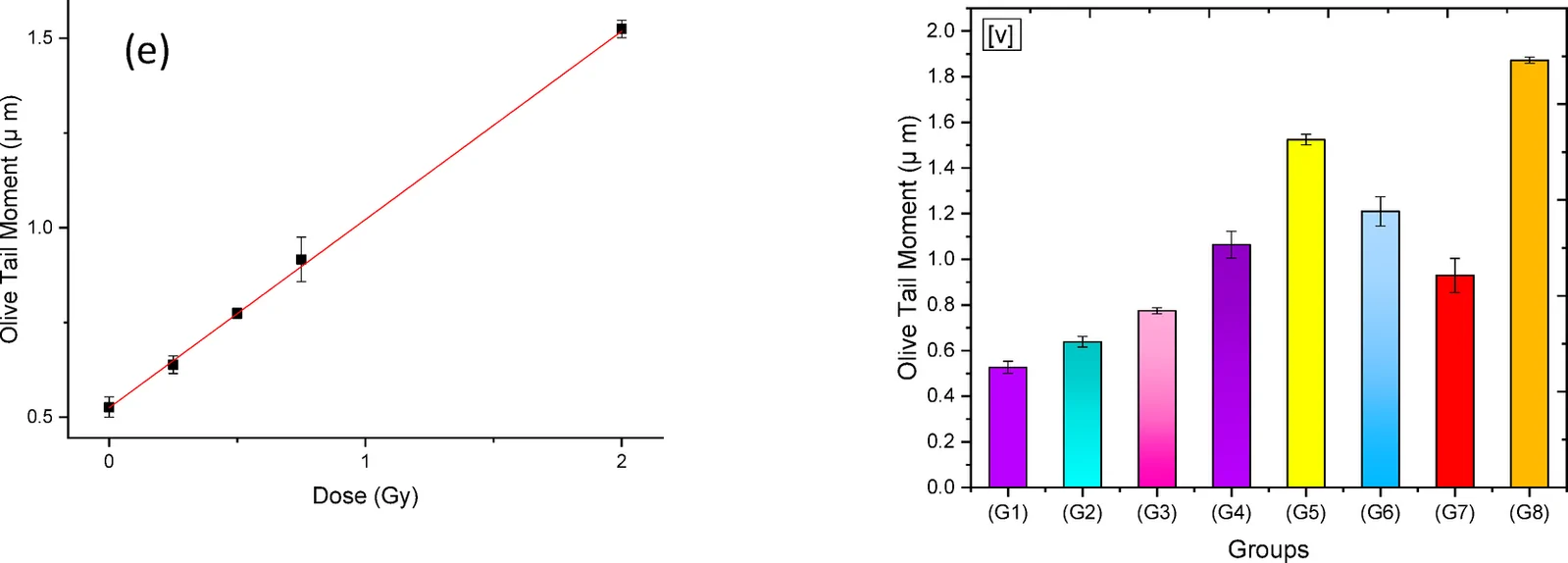
Effects of different acute gamma radiation doses (0, 0.25, 0.50, 0.75, and 2 Gy) on comet assay parameters across the five groups (G1–G5). Panels (**a**–**e**) represent [CA], [TL], [DNA%], [TM], and [OTM], respectively. Panels (**i**–**v**) illustrate the overall trends of these parameters across all groups.

For the irradiated groups, Fig. 2 shows the dose-response of Comet Assay Score (CAS), Tail Length (TL), DNA percentage of tail (DNA%), Tail Moment (TM), and Olive Tail Moment (OTM). For every measured component of the CAS method, Table 3 displays the fitting parameters of the acute Gamma radiation dose response. This image makes it evident that all CAS parameters for liver cells showed an increase in the groups subjected to gamma doses of 0.25 Gy (G2), 0.50 Gy (G3), 0.75 Gy (G4), and 2 Gy (G5). For the CAS, the dose-response behavior was parabola, rising from 6 to 16.5%. Tail Length dramatically in a linear response, from 4.7 to 7.46 μm. DNA% is a ExpGro fitting, while TM is a parabola fitting, the dose-response behavior rising from 11.815 to 17.76% and from 1.166 to 3.78 μm, respectively. As the gamma increases OTM climb linearly fitted and 0.53 to 1.524 μm, respectively. Comparing the 0.25 + 2 Gy (G6) and 0.50 + 2 Gy (G7) groups with the control group, all CAS parameters showed an increase, including CA% (15.516% and 11.46%), tail length (TL) (6.57 and 5.61 μm), DNA% (17.09% and 15.65%), tail moment (TM) (2.75 and 2.16 μm), and olive tail moment (OTM) (1.21 and 0.93 μm), respectively. In contrast, when comparing the 2 Gy-only group (G5) with the same pre-exposed groups (G6 and G7), a clear radio-adaptive response (RAR) pattern was observed. The percentage differences were as follows: CA% (5.85% and 30.42%), TL (11.95% and 24.75 μm), DNA% (3.76% and 11.88%), TM (27.37% and 42.82 μm), and OTM (20.61% and 39.05 μm), respectively. In contrast to the G5 group, the group exposed to 0.75 + 2 Gy (G8) demonstrated an increase in all CAS parameters, including CA% (7.52), TL (3.28%), DNA% (9.89), TM (7.49%), and OTM (22.8%), showing ongoing damage from gamma radiation exposure. DNA% was selected as the primary endpoint for quantifying DNA damage, as it provides a direct and widely accepted measure of DNA migration in comet assays.

**Table 3:** The fitting parameters of the relation between dose and comet assay for the groups that were exposed to acute doses of gamma radiation in addition to the control (zero doses) group.

Parameter	Type of fitting	Equation	Equation constants	*R*-value	Significance
CA%	Parabola	y = A + B*x + C*x^2	$\:A=5.75729\:\pm\:\:0.3268$ $\:B=12.2167\:\pm\:\:1.0986$ $\:C=-3.46237\:\pm\:\:0.521$	* 0.99459*	9.1349$\:{\times\:10}^{-4}$
TL	Linear	y = a + b*x	$\:a=4.85\pm\:\:0.017$ $\:b=1.284\pm\:\:0.023$	* 0.9991*	$\:1.19\times\:{10}^{-5}$
DNA%	ExpGro	$\:={\:y}_{0}+\:{A}_{1}\:{e}^{(x/t1)}$	$\:{\:y}_{0}=17.74115\:\pm\:\:0.32525$ $\:{A}_{1}=-5.95423\:\pm\:\:0.40112$ $\:{t}_{1}=-0.4015\:\pm\:\:0.06658$	* 0.98942*	$\:5.75\times\:{10}^{-5}$
TM	Parabola	y = A + B*x + C*x^2	$\:A=1.1087\:\pm\:\:0.09561$ $\:B=2.02195\:\pm\:\:0.25428$ $\:C=-0.59896\:\pm\:\:0.11709$	* 0.98576*	* 0.00154*
OTM	Linear	y = a + b*x	$\:a=0.52218\pm\:\:0.00414$ $\:b=0.5016\pm\:\:0.00452$	* 0.99976*	$\:3.191\times\:{10}^{-7}$

### RAR equivalent dose (RARED) and RAR factor (RARF)

Equation 1 is used to calculate RARF values, which are then compiled in Table 4. The values of RARED were approximated from the calibration curve used for the fitting of measured marker data points. For DNA% and OTM parameters, the mean values of these RAR equivalent doses for 0.25 + 2 Gy range from 0.89 to 1.45 Gy, with an average of 1.17 ± 0.19 Gy (σ = 1), whereas the values for 0.50 + 2 Gy range from 0.52 to 0.81 Gy, with an average of 0.64 ± 0.09 Gy. However, the RARF values, Table 4 show OTM and DNA% values for the 0.25 + 2 Gy range from 0.50 to 0.72, with an average of 0.61 ± 0.09. While an average of 0.944 ± 0.04 for 0.5 + 2 Gy, ranging from 0.87 for OTM to 0.992 for DNA%. These findings offer insightful information for more research and interpretation.

**Table 4:** The mean of the equivalent dose of RAR and the calculated factor of RAR for each marker under the study as an acute Gamma radiation dose for 0.25 + 2 and 0.5 + 2 Gy doses.

Mean of RARED (Gy) and RARF													
Dose	Parameters	GssG	GSH	GssG/GSH ratio	CAT	SOD	CA	TL	DNA%	TM	OTM	Average	SD
(0.25 + 2 Gy)	RARED	1.03	1.06	1.012	1.45	1.01	1.22	1.004	0.89	1.34	1.38	1.14	0.19
	RARF	0.65	0.64	0.66	0.63	0.69	0.51	0.66	0.72	0.66	0.50	0.61	0.09
(0.50 + 2 Gy)	RARED	0.77	0.61	0.58	0.70	0.59	0.56	0.61	0.58	0.61	0.81	0.64	0.086
	RARF	0.89	0.96	0.97	0.93	0.97	0.96	0.95	0.96	0.97	0.87	0.944	0.035

## Discussion

Antioxidant enzymes are essential for shielding cells from the damaging effects of free radicals in the context of radiation exposure^47^. These enzymes protect organisms from oxidative harm by eliminating reactive oxygen species (ROS)^48^. Cell differentiation, growth regulation, immunity, programmed cell death, and defense against Microorganisms exhibit biological activities that depend on reactive oxygen species (ROS)^49–51^. Numerous ROS, including superoxide, hydrogen peroxide, and hydroxyl radicals, are produced by ionizing radiation and can seriously harm proteins, lipids, and nucleic acids and damaged mitochondria be also major intracellular sources of ROS. Moreover, the lipid component of the membrane makes radiation damage to lipids more severe^52^. The body’s defense systems against oxidative stress include antioxidant mechanisms, DNA repair pathways, and immune responses. These systems collectively mitigate cellular damage induced by free radicals. Exposure to ionizing radiation increases free radical production in a dose-dependent manner, leading to depletion of antioxidant reserves and enhanced cellular and tissue injury^53^. Therefore, maintaining a balance between oxidants and antioxidants is essential for cell survival and overall health^54^. Excessive oxidative stress resulting from impaired antioxidant defenses is associated with increased health risks in humans^54^.

This study examined how rats’ susceptibility to oxidative stress was affected by whole-body exposure to modest amounts of gamma-ray radiation^55^. Following radiation exposure, a markedly lower level of GSH content was discovered, suggesting problems with overall oxidative stress, but they could also be linked to problems with lipid metabolism. We looked at the enzyme activity to confirm their roles in the adaptive response. The findings are quite intriguing when considering oxidative cellular reactions brought about by low or moderate exposure to ionizing radiation and adaptive response^56^. The current study’s findings demonstrated a low production of antioxidant enzymes, which can be fitted with a polynomial in GSH, a cubic fitting in CAT and SOD, a linear fitting in GSSG and the ratio of GSSG/GSH in acute gamma radiation exposure for both 0.25, 0.50, and 0.75 Gy. When compared to 2 Gy (G5), which showed an adaptive response in rat blood cells, exposure to 0.50 + 2 Gy (G7) of gamma dose produced increased quantities of the antioxidant enzymes GSSG, GSH, CAT, and SOD along with a decrease in free radicals. GSSG, GSH, CAT, and SOD had RARED values of 0.77, 0.61, 0.68, and 0.58 Gy and RARF values of 0.89, 0.96, 0.93, and 0.97, respectively. Since early increases in enzyme activity following low-dose ionizing radiation exposure have been shown to occur in the absence of changes in transcription^57^. The observed increases in antioxidant enzyme activities at 2 Gy (e.g., GSH, CAT, and SOD) may reflect post-transcriptional regulation or the mobilization of pre-existing enzyme pools rather than de novo synthesis. Rather than representing a true adaptive upregulation of enzyme expression per se, this early response may be attributed to radiation-induced oxidative stress leading to conformational modifications and the activation of latent enzymatic reserves, ultimately contributing to cellular protection against oxidative damage.

A flexible, simple, and adaptive method for assessing DNA damage and repair at the cellular level is the CAS. After brief exposure, the CA enables identification of early or acute DNA damage, which may be repaired or lead to mutations and/or programmed cell death (apoptosis), resulting in less noticeable DNA damage^58^. Free radicals, such as hydroxyl radicals, lipid peroxide radicals, superoxide radicals, and lipid radicals, have also been linked to radiation-induced cell damage. In addition to direct DNA damage, long-lived lipid radicals contribute to damage of biological membranes, leading to a variety of biological problems^55^. The purine and pyrimidine bases, as well as the deoxyribose backbone, are harmed when the hydroxyl radical interacts with every part of the DNA molecule^11,12,14^. Elmarakby et al., (2021)^59^ A comet assay study conducted in mice investigated gamma radiation doses and identified dose-dependent increases in DNA damage. In comparison, our data from rats exposed to a 2 Gy challenge dose demonstrated significantly reduced DNA damage when preceded by low priming doses, suggesting a clear adaptive response (RAR)^60^. It should be highlighted, nonetheless, that significant efforts have already been made to standardize the comet assay score (CAS) for evaluating hazards to human health, as the assay’s measurement of DNA damage in circulating leukocytes may serve as a predictor of mortality risk. International databases and standardized reporting frameworks, such as the Minimum Information for Reporting Comet Assay (MIRCA) guidelines, provide baseline DNA damage levels and help account for confounding factors^43,61^.

The evaluation of adaptive responses using the comet assay may gain increasing importance in biodosimetry and in assessing potential health risks associated with ionizing radiation, including radiotherapy and occupational exposure, where chromosomal aberrations have traditionally been used. In the present study, all comet assay parameters (TM, DNA%, TL, and OTM) increased following acute gamma radiation exposure. When compared to the irradiated groups with a challenge dosage of 2 Gy (G6 and G7), low-dose irradiated groups (G2 and G3) displayed less DNA damage. The adaptive response was shown when exposed to gamma radiation for 0.50 Gy before the 2 Gy challenge dose, while it is less evident when exposed to gamma radiation for 0.25 Gy prior to the 2 Gy challenge dosage. Following the 2 Gy challenge dose, rats previously exposed to low-dose-rate gamma radiation exhibited a clear adaptive response in the comet assay, as indicated by reduced final DNA damage levels. When compared to 2 Gy (G5), exposure to 0.50 + 2 Gy (G7) of gamma dose produced decreased quantities of the CAS parameters, such as CA, DNA%, and TM. These parameters had RARED values of 0.60, 0.52, and 0.57 Gy and RARF values of 0.96, 0.992, and 0.97, respectively. In general, the average percentage of DNA in the tail was 0.89% in group G6 compared to group G5. DNA damage was more serious in group G6, while it was lower in group G7. This indicates that group G6 exhibited more DNA damage than group G7. This suggests that group 6 has more DNA movement than group 7. It is important to note that the results of the CAS with gamma radiation are different from those in our earlier study using thermal neutrons^59^. This is attributed to differences in radiation quality, as neutrons induce more complex DNA damage due to their higher linear energy transfer (LET). In the comet assay, this results in increased DNA strand breaks, reflected in a higher tail moment and DNA%. These findings emphasize that radiation type and quality significantly influence the extent of DNA damage and cellular responses.

Following pre-exposure to 0.25 and 0.5 Gy, there was a decrease in GSSG levels, an increase in CAT activity, and a decrease in DNA damage. These findings suggest that a radioadaptive response (RAR) was induced, most likely through strengthening antioxidant defenses and enhancing DNA repair mechanisms. The decrease in GSSG indicates a reduced oxidative state and suggests improved redox balance, which points to better oxidative balance. The lack or reversal of this effect at 0.75 Gy is consistent with a biphasic hormetic response, suggesting the existence of a dose-dependent threshold beyond which oxidative stress may exceed cellular defense capacity^27,28^. Our findings are consistent with earlier studies demonstrating that low-dose radiation priming can trigger an adaptive response: in human A549 cells, a 0.5 Gy pre-treatment decreased apoptosis and altered inflammatory and antioxidant markers^62^ in human peripheral blood mononuclear cells, gamma radiation triggered antioxidant pathways such as Nrf2^56^.

Finally, curve fitting analysis showed that GSH followed a polynomial regression model, whereas GSSG, the GSSG/GSH ratio, TL, and OTM exhibited linear regression patterns. In contrast, DNA% responses were best described by an ExpGro model. CAS, CAT, and TM demonstrated a parabolic fit in response to low-dose gamma radiation, while SOD showed a characteristic dose–response fitting curve. The ideal option for the bio dosimeters is any linearly fitted parameter with an R-value higher than 0.99. OTM the most accurate biomarker, although not as precise. A thorough understanding of the adaptive response and dosimetry for low doses of gamma radiation, including the length of the intervals between radiation exposures, long-term stability, and the storage temperature effect, still needs additional research.

It is important to recognize the various limitations of this study. This study was limited to a relatively small number of radiation dose levels used for curve fitting, which may affect statistical power. Therefore, it was restricted in its ability to fully characterize dose–response relationships. Second, the experimental paradigm only contained male Wistar rats, which limited the direct extrapolation of the results to human populations and did not allow full generalization of the findings. Furthermore, the study did not assess the underlying molecular pathways causing the reported effects; instead, it focused exclusively on a limited number of biochemical markers. To fully understand the processes behind the radioadaptive response, Future research should consider larger animal cohorts, human studies, sex-specific responses, and a broader range of radiation doses, combined with molecular-level investigations.

## Conclusion

Long-term low-level gamma radiation exposure can lead to increased DNA damage and a reduction in antioxidants including GSH, SOD, and CAT. GSSG, the ratio of GSSG/GSH, TL, and OTM concentration have all been found to be biomarkers for evaluating acute low-dose gamma radiation in the current study; OTM is the most useful biomarker for acute exposure.

Furthermore, rats who received 0.25–0.50 Gy prior to a 2 Gy gamma radiation challenge showed immune system activation, indicating a quick two-week recovery from radiation effects. This discovery is supported by higher antioxidant concentrations, fewer free radicals, and less DNA damage, as seen by lower DNA% and tail moment. These results demonstrate that the radioadaptive response is dose-dependent and takes place within a low-dose range, after which cellular defensive mechanisms are activated. This highlights how crucial it is to precisely determine the ideal priming dosage when examining adaptive response to ionizing radiation. The results obtained are of great interest in the context of oxidative cellular responses induced by low-dose ionizing radiation exposure and adaptive responses. The calculated RAR factor indicated that the 0.5 Gy priming dosage induced a stronger adaptive response, while the projected average RARED for compounded doses of 0.25 + 2 Gy and 0.5 + 2 Gy was roughly 52% and 75% of the corresponding true doses. The effects of acute gamma doses are the driving force of our investigation of combined gamma and neutron exposures. However, biological systems continue to be exposed and to suffer from ionizing radiation exposures. Therefore, to avoid the negative effects of ionizing radiation exposure, we highly advise proper management of radioactive materials in accordance with international radiation safety rules.

## Data Availability

The datasets generated and/or analyzed during the current study are available from the corresponding author on reasonable request.

## References

[1] Abend, M. et al. Examining radiation-induced in vivo and in vitro gene expression changes of the peripheral blood in different laboratories for biodosimetry purposes: First RENEB gene expression study. *Radiat. Res.* **185** (2), 109–123. 10.1667/RR14221.1.2 (2016). 26829612

[2] Azqueta, A. et al. Technical recomendations to perform the alkaine standard and enzyme-modified comet assay in huan biomonitoring studies. *Mutat. Re*. **843**, 24–32 (2019). 10.1016/j.mrgentox.2019.04.007 31421734

[3] Blakely, W. F., Prasanna, P. G., Grace, M. B. & Miller, A. C. Radiation exposure assessment using cytological and molecular biomarkers. *Radiat. Prot. Dosimetry*. **97**, 17–23 (2001). 11763353 10.1093/oxfordjournals.rpd.a006633

[4] Hagmar, L., Stromberg, U., Tinnerberg, H. & Mikoczy, Z. The usefulness of cytogenetic biomarkers as intermediate endpoints in carcinogenesis. *Int. J. Hyg. Environ. Health*. **204**, 43–47 (2001). 11725344 10.1078/1438-4639-00071

[5] Thierens, H. et al. Chromosomal radiosensitivity study of temporary nuclear workers and the support of the adaptive rsponse induced by occupational exposure. *Int. J. Radiat. Biol.* **78**, 1117–1126 (2002). 12556339 10.1080/0955300021000034710

[6] Li-Ping Ma, Chen, J. et al. Biodosimetry based on gamma-H2AX quantification in human peripheral blood lymphocytes after partial-body irradiation. *Health Phys.* **126** (3), 134–140. 10.1097/HP.0000000000001779 (2024). 38117190

[7] Kanagaraj, K. et al. BAX and DDB2 as biomarkers for acute radiation exposure in the human blood ex vivo and non-human primate models. *Sci. Rep.* **14**, 19345. 10.1038/s41598-024-69852-z (2024). 39164366 PMC11336173

[8] Grace, M. B., McLeland, C. B. & Blakely, W. F. Real-time quantitative RT-PCR assay of GADD45 gene expression changes as a biomarker for radiation biodosimetry. *Int. J. Radiat. Biol.* **78**, 1011–1021 (2002). 12456288 10.1080/09553000210158056

[9] Pellmar, T. C. & Rockwell, S. Priority list of research areas for radiological nuclear threat countermeasures. *Radiat. Res.* **163**, 115–123 (2005). 15606315 10.1667/rr3283

[10] Touil, N., Aka, P. V., Buchet, J. P., Thierens, H. & Kirsch-Volders, M. Assessment of genotoxic effects related to chronic low-level exposure to ionizing radiation using biomarkers for DNA damage and repair. *Mutagenesis* **17**, 223–232 (2002). 11971994 10.1093/mutage/17.3.223

[11] Fairbairn, D. W., Olive, P. L. & O’Neill, K. L. The comet assay: A comprehensive review. *Mutat. Res.* **339** (1), 37–59. 10.1016/0165-1110(94)00013-3 (1995). 7877644

[12] Olive, P. L., Banáth, J. P. & Durand, R. E. Heterogeneity in radiation-induced DNA damage and repair in tumor and normal cells measured using the comet assay. *Radiat. Res.* **122** (1), 86–94 (1990). 2320728

[13] Verde, P. E. et al. Application of public-domain statistical analysis software for evaluation and comparison of comet assay data. *Mutat. Res.* **604**, 71–82 (2006). 16540366 10.1016/j.mrgentox.2006.01.002

[14] Cadet, J. et al. Radiationinduced DNA damage: Formation, measurement, and biochemical features. *J. Environ. Pathol. Toxicol. Oncol.* **23**, 33–43 (2004). 14994993 10.1615/jenvpathtoxoncol.v23.i1.30

[15] Popp, W. et al. Biomarkers of genetic damage and inflammation in blood and bronchoalveolar lavage fluid among former German uranium miners: A pilot study. *Radiat. Environ. Biophys.* **39**, 275–282 (2000). 11200971 10.1007/s004110000072

[16] Garaj-Vrhovac, V. & Kopjar, N. The alkaline comet assay as biomarker in assessment of DNA damage in medical personnel occupationally exposed to ionizing radiation. *Mutagenesis* **18**, 265–271 (2003). 12714692 10.1093/mutage/18.3.265

[17] Halliwell, B. & Gutteridge, J. M. C. *Free Radicals in Biology & Medicine. *5th edn (Oxford Universityy, 2015).

[18] Lobo, V., Patil, A., Phatak, A. & Chandra, N. Free radicals, antioxidants and functional foods: Impact on human health. *Pharmacogn. Rev.* **4** (8), 118–126. 10.4103/0973-7847.70902 (2010). 22228951 PMC3249911

[19] Valko, M. et al. Free radicals and antioxidants in normal physiological functions and human disease. *Int. J. Biochem. Cell Biol.* **39** (1), 44–84. 10.1016/j.biocel.2006.07.001 (2007). 16978905

[20] Ayala, A., Muñoz, M. F. & Argüelles, S. Lipid peroxidation: Production, metabolism, and signaling mechanisms of malondialdehyde and 4-hydroxy-2-nonenal. *Oxidative Med. Cell. Longev.* **2014**, 360438. 10.1155/2014/360438 (2014). PMC4066722 24999379

[21] Durak, D., Calendar, S., Uzun, F. G., Demir, F. & Calendar, Y. Mercury chloride-induced oxidative stress in human erytrocytes and the effect of vitamin C and E in vitro. *Afr. J. Biotechnol.* **9**, 488–495 (2010).

[22] Winarsi, H. *Natural Antioxidants and Free Radicals* 3 edn (Kanisius, 2007).

[23] Murray, R. K., Granner, D. K. & Roadwell, V. W. Harper’s Illustrated Biochemistry. In: (eds Wulandari, N., Randy, L., Dwijayanti, L., Liena, Dany, F. & Rachman, L. Y.) Harper Biochemistry. 27 th ed. Jakarta: EGC. (2009).

[24] Mortazavi, S. et al. Increased radio resistance to lethal doses of gamma rays in mice and rats after exposure to microwave radiation emitted by a GSM mobile phone simulator. *Dose Response: Publ. Int. Hormesis Soc.* **11** (2), 281–292. 10.2203/dose-response.12-010.Mortazavi (2012). PMC3682203 23930107

[25] Olivieri, G., Bodycote, J. & Wolff, S. Adaptive response of human lymphocytes to low concentrations of radioactive thymidine. *Science* **223** (4636), 594–597 (1984). 6695170 10.1126/science.6695170

[26] Azzam, E., Raaphorst, G. & Mitchel, R. Radiation-induced adaptive response for protection against micronucleus formation and neoplastic transformation in C3H 10T1/2 mouse embryo cells. *Radiat. Res.* **138** (1s), S28–S31 (1994). 8146320

[27] Calabrese, E. J. Preconditioning is hormesis part I: Documentation, dose-response features and mechanistic foundations. *Pharmacol. Res.* **110**, 242–264 (2016). 26757428 10.1016/j.phrs.2015.12.021

[28] Abdelgawad, M. H., Awad, M. M., Eraba Khairy, M. T. & Egypt Study of the Effects of Naturally Occurring Radioactive Materials on Blood Indices in Blood’s Rats. *J. Biophys. Biomed. Eng. Vol* **20**, 1–7, DOI: 10.21608/EJBBE.2019.7930.1024 (2019).

[29] Joiner, M. C., Lambin, P. & Marples, B. Adaptive response and induced resistance. *C R Acad. Sci. III*. **322** (2–3), 167–175 (1999). 10196669 10.1016/s0764-4469(99)80040-7

[30] Bugała, E. & Fornalski, K. W. Radiation adaptive response for constant dose-rate irradiation in high background radiation areas. *Radiat. Environ. Biophys.* **64**, 1–16 (2024). 10.1007/s00411-024-01093-0 PMC11971215 39470814

[31] Taheri, M. et al. Evaluation of the effect of radiofrequency radiation emitted from wi-fi router and mobile phone simulator on the antibacterial susceptibility of pathogenic bacteria Listeria monocytogenes and *Escherichia coli*. *Dose Response*. **15** (1), 1559325816688527 (2017). 28203122 10.1177/1559325816688527 PMC5298474

[32] Mokarram, P., Sheikhi, M., Mortazavi, S., Saeb, S. & Shokrpour, N. Effect of exposure to 900 MHz GSM mobile phone radiofrequency radiation on estrogen receptor methylation status in colon cells of male sprague dawley rats. *J. Biomed. Phys. Eng.* **7** (1), 79–86 (2017). 28451581 PMC5401136

[33] Tapio, S. & Jacob, V. Radioadaptive response revisited. *Radiat. Environ. Biophys.* **46**, 1–12 (2007). 17131131 10.1007/s00411-006-0078-8

[34] El-Marakby, S. M., Awad, M. M., Eraba, K. M., Abdelgawad, M. H. & Desouky, O. S. Assessment of chronic exposure effects and radioadaptive response of natural occurring radioactive materials (NORM). *Radiat. Phys. Chem.* **166**, 108502 (2020).

[35] Awad, M. M. et al. Biomarker dosimetry of acute low level of thermal neutrons and radiation adaptive response effect on rats. *Sci. Rep.* **14**, 18534. 10.1038/s41598-024-68640-z (2024). 39122766 PMC11316017

[36] Attia, A. M. M., Aboulthana, W. M., Hassan, G. M. & Aboelezz, E. Assessment of absorbed dose of gamma rays using the simultaneous determination of inactive hemoglobin derivatives as a biological dosimeter. *Radiat. Environ. Biophys.* **59** (2020). 10.1007/s00411-019-00821-1 31734721

[37] Moheban, A. A., Chang, H. H. & Havton, L. A. The suitability of propofol compared with urethane for anesthesia during urodynamic studies in rats. *J. Am. Assoc. Lab. Anim. Sci.* **55** (1), 89–94 (2016). 26817985 PMC4747016

[38] Zatroch, K. K., Knight, C. G., Reimer, J. N. & Pang, D. S. Refinement of intraperitoneal injection of sodium pentobarbital for euthanasia in laboratory rats (Rattus norvegicus). *BMC Vet. Res.* **13** (1), 60. 10.1186/s12917-017-0982-y (2017). 28222732 PMC5320784

[39] Sinha, A. K. Colorimetric assay of catalase. *Anal. Biochem.* **47**, 389–394 (1972). 4556490 10.1016/0003-2697(72)90132-7

[40] Minami, M. & Yoshikawa, H. A simplified assay method activity for clinical use of superoxide dismutase. *Clin. Chim. Acta*. **92**, 337–342 (1979). 436274 10.1016/0009-8981(79)90211-0

[41] Ahmed, A. E., Hussein, G. I. & Loh, J. Abdel-rahman, S. Z. Studies on the mechanism of haloacetonitrile-induced gastrointestinal toxicity: Interaction of dibromoacetonitrile with glutathione and glutathione-s-transferase in rats. *J. Biochem. Toxicol.* **6**, 115–121 (1991). 1941897 10.1002/jbt.2570060205

[42] Wu, G., Fang, Y. Z., Yang, S., Lupton, J. R. & Turner, N. D. Glutathione metabolism and its implications for health. *J. Nutr.* **134** (3), 489–492. 10.1093/jn/134.3.489 (2004). 14988435

[43] Møller, P. et al. Minimum information for reporting on the comet assay (MIRCA): Recommendations for describing comet assay procedures and results. *Nat. Protoc.* **15** (12), 3817–3826. 10.1038/s41596-020-0398-1s (2020). 33106678 PMC7688437

[44] Singh, N. P., McCoy, M. T., Tice, R. R. & Schneider, E. L. A simple technique for quantitation of low levels of DNA damage in individual cells. *Exp. Cell. Res.* **175**, 184–191 (1988). 3345800 10.1016/0014-4827(88)90265-0

[45] Yonezawa, M., Takeda, A. & Misonoh, J. Acquired radioresistance after low dose X-irradiation in mice. *J. Radiat. Res.* **31**, 256–262 (1990). 2246750 10.1269/jrr.31.256

[46] Fornalski, K. W., Adamowski, Ł., Dobrzyński, L. & Jarmakiewicz, R. The radiation adaptive response and priming dose influence: The quantification of the Raper–Yonezawa effect and its three Parameter model for postradiation DNA lesions and mutations. Radiat. *Environ. Biophys.* **61**, 221–239 (2022). 10.1007/s00411-022-00963-9 PMC9021059 35150289

[47] Gad, N. S. Oxidative stress and antioxidant enzymes in *Oreochromis niloticus* as biomarkers of exposure to crude oil pollution Nahed S. *Gad. Int. J. Environ. Sci. Eng.* **1**, 49–58 (2011).

[48] Yin, G. Y., Yin, Y. F. & He, X. F. Effect of zhuchun pill on immunity and endocrine function of elderly with kidney-yang deficiency. *Zhongguo Zhong xi yi jie he za zhi Zhongguo Zhongxiyi jiehe zazhi Chin. J. Integr. Tradit West. Med.* **15**, 601–603 (1995). 8704426

[49] Ghosh, J. & Myers, C. E. Inhibition of arachidonate 5-lipoxygenase triggers massive apoptosis in human prostate cancer cells. *Proc. Natl. Acad. Sci. U. S. A.* **95**, 13182–13187 (1998). 10.1073/pnas.95.22.13182 PMC23752 9789062

[50] Lee, Y. J. et al. Glucose deprivation-induced cytotoxicity and alterations in mitogen-activated protein kinase activation are mediated by oxidative stress in multidrug-resistant human breast carcinoma cells. *J. Biol. Chem.* **273**, 5294–5299 (1998). 9478987 10.1074/jbc.273.9.5294

[51] Chevion, S., Or, R. & Berry, E. M. The antioxidant status of patients subjected to today body irradiation. **47**, 1069–1077 (2008). 10.1080/15216549900202143 10410248

[52] Kohen, R. & Nyska, A. Oxidation of biological systems: Oxidative stress phenomena, antioxidants, redox reactions, and methods for their quantification. *Toxicol. Pathol.* **30**, 620–650 (2002). 12512863 10.1080/01926230290166724

[53] Awd, M. M., Abdelgawad, M. H., Elsayed, E. A. & Ereiba, K. T. Investigation of the impact of thermal acute low-level neutron radiation on various hematological parameters and lipid profiles in Rats. *Egypt. J. Biomed. Eng. Biophys.* **25** (1), 75–86 (2024).

[54] Schols, A. M. et al. Nutritional assessment and therapy in COPD: A European respiratory society statement. *Eur. Respir J.* **44**, 1504–1520 (2014). 25234804 10.1183/09031936.00070914

[55] Kumaravel, T. S. & Jha, A. N. Reliable comet assay measurements for detecting DNA damage induced by ionising radiation and chemicals. *Mutat. Research-Genetic Toxicol. Environ. Mutagen.* **605** (1–2), 7–16. 10.1016/j.mrgentox.2006.03.002 (2006). 16621680

[56] Hardmeier, R., Hoeger, H., Fang-Kircher, S., Khoschsorur, A. & Lubec, G. Transcription and activity of antioxidant enzymes after ionizing irradiation in radiation-resistant and radiation-sensitive mice. *Proc. Natl. Acad. Sci. U S A*. **94** (14), 7572–7576. 10.1073/pnas.94.14.7572 (1997). 9207133 PMC23863

[57] Upadhyay, M. et al. Identification of plasma lipidome changes associated with low dose space-type radiation exposure in a murine model. *Metabolites* **10** (6), 252. 10.3390/metabo10060252 (2020). 32560360 PMC7345467

[58] Collins, A. R. Measuring oxidative damage to DNA and its repair with the comet assay. *Biochim. et Biophys. Acta (BBA) - Gen. Subj.* **1840** (2), 794–800. 10.1016/j.bbagen.2013.04.022 (2014). 23618695

[59] El-Marakby, S., Abdelgawad, M., Awd, M., Eraba, K. & Desouky, O. DNA damage detection after chronic exposure and radioadaptive response of naturally occurring radioactive materials (NORM). *Arab. J. Nucl. Sci. Appl.* **54** (3), 34–45 (2021).

[60] Ali, Y. F., Hassan, I. M., Abdelhafez, H. M. & Desouky, O. S. 0.5 Gy confers resistance to a subsequent high dose of γ-rays by modulating HO-1/Nrf2 and apoptosis pathways. *Sci. Rep.* **15** (1), 9199. 10.1038/s41598-025-91667-9 (2025). 40097469 PMC11914414

[61] Møller, P. et al. Minimum information for reporting on the comet assay (MIRCA): Recommendations for describing comet assay procedures and results. *Nat. Protoc.* **15** (12), 3817–3826. (2020). 10.1038/s41596-020-0398-1s 33106678 PMC7688437

[62] Paraswania, N., Thohb, M., Bhilwadeb, H. N. & Ghosha, A. Early antioxidant responses via the concerted activation of NF-κB and Nrf2 characterize the gamma-radiation-induced adaptive response in quiescent human peripheral blood mononuclear cells. *Mutat. Res. Gen. Tox En*. **831**, 50–61 (2018). 10.1016/j.mrgentox.2018.04.007 29875077

